# The GDSL-Lipolytic Enzyme Lip1 Is Required for Full Virulence of the Cucurbit Pathogenic Bacterium *Acidovorax citrulli*

**DOI:** 10.3390/microorganisms10051016

**Published:** 2022-05-12

**Authors:** Tally Rosenberg, Irene Jiménez-Guerrero, Dafna Tamir-Ariel, Tali Yarnitzky, Saul Burdman

**Affiliations:** 1 Department of Plant Pathology and Microbiology, Institute of Environmental Sciences, The Robert H. Smith Faculty of Agriculture, Food and Environment, The Hebrew University of Jerusalem, Rehovot 7610001, Israel; tally.rosenberg@gmail.com (T.R.); ijimgue@us.es (I.J.-G.); dafnata@savion.huji.ac.il (D.T.-A.); yarnitzky.tali@gmail.com (T.Y.); 2 Departamento de Microbiología, Facultad de Biología, Universidad de Sevilla, 41012 Sevilla, Spain

**Keywords:** *Acidovorax citrulli*, bacterial fruit blotch, lipase, esterase, virulence, type IV pili, twitching motility, type II secretion

## Abstract

Bacterial fruit blotch caused by *Acidovorax* *citrulli* is a serious disease of cucurbit crops. Here we report characterization of a mutant strain of *A. citrulli* M6 defective in *lip1*, a gene encoding a lipolytic enzyme. The M6-*lip1^-^* mutant was detected in a mutant library screen aimed at identifying M6 mutants with altered levels of twitching motility. In this screen M6-*lip1^-^* was the only mutant that showed significantly larger twitching motility haloes around colonies than wild-type M6. Sequence analyses indicated that *lip1* encodes a member of the GDSL family of secreted lipolytic enzymes. In line with this finding, lipolytic assays showed that the supernatants of M6-*lip1^-^* had lower lipolytic activity as compared with those of wild-type M6 and a *lip1*-complemented strain. The mutant was also affected in swimming motility and had compromised virulence on melon seedlings and on *Nicotiana benthamiana* leaves relative to wild-type and complemented strains. Lip1 contains a predicted N-terminal signal sequence for type II secretion. Evidence from our study confirms Lip1 is indeed secreted in a type II secretion-dependent manner, and this is required for full virulence of *A. citrulli*. To the best of our knowledge this is the first study reporting contribution of lipolytic activity to virulence of a plant-pathogenic *Acidovorax* species.

## 1. Introduction

The Gram-negative bacterium *Acidovorax citrulli* belongs to the *Betaproteobacteria* class and causes bacterial fruit blotch (BFB), a threatening disease of cucurbits worldwide, and mainly of melon and watermelon [1]. *Acidovorax citrulli* is a seed borne pathogen and seed transmission has been responsible for the fast spread of BFB to many parts of the world [2]. The bacterium infects all aerial parts of the plant and at all developmental stages, from the young seedling to the fruit. To date, there are no reliable sources of BFB resistance and the available disease management strategies have limited efficacy [2,3]. Based on genetic and biochemical features, *A. citrulli* can be divided into two main groups that also differ in terms of host preference: while group I strains were mainly isolated from melon and are moderately to highly aggressive to this plant, and to a wide range of cucurbit species, most group II strains were isolated from watermelon, are highly aggressive to this plant, and only mildly so to other cucurbits [4,5,6,7].

The successful establishment of a pathogenic bacterium in the host tissue requires the coordinated expression of virulence factors. Similarly to several Gram-negative pathogenic bacteria, *A. citrulli* relies on a functional type III secretion system and type III-secreted effector proteins for pathogenicity [8,9,10]. Type IV pili (T4P) also play an important role in *A. citrulli* virulence as well as in surface adhesion, biofilm formation and colonization [11,12]. T4P are hair-like appendages found on the poles of the cell surface. They mediate twitching motility, which is an effective flagellum-independent form of bacterial surface motility [13].

*Acidovorax citrulli* M6 was isolated from a BFB outbreak of melon in Israel in 2002 [6] and in the following years became a model group I strain for fundamental and applied investigation of BFB. In 2016, we reported the draft sequence of the M6 genome [14], and three years later we were able to fully assemble the genome of this strain using single molecule real time (SMRT; PacBio) sequencing [15]. We recently screened an EZ-Tn5 mutant library with ~10,000 mutants in the background of strain M6 [11] for mutants altered in twitching motility. This screen revealed fifty twitching defective mutants that were disrupted in twenty different genes. These included genes involved in T4P biogenesis, regulation and chemotaxis [16]. In agreement with previous findings supporting an important contribution of T4P to *A. citrulli* virulence [11], all mutants were significantly impaired in this trait [16].

In the aforementioned screen, we also identified a mutant that produced significantly larger twitching haloes around bacterial colonies as compared with wild-type M6. Sequence analysis of this mutant revealed that the transposon insertion disrupted a gene encoding a lipolytic enzyme. Lipolytic enzymes have been extensively studied due to the high interest for biotechnological applications [17,18,19]. However, only relatively few studies have dealt with their contribution to virulence of phytopathogenic bacteria. With that said, several lipases and esterases were shown to be important virulence factors in some plant-pathogenic bacteria [20,21,22,23,24,25]. The objective of this study was to assess the contribution of this lipolytic enzyme, which we named Lip1, to the virulence and other traits of *A. citrulli*.

## 2. Materials and Methods

### 2.1. Bacterial Strains and Plasmids

Bacterial strains and plasmids used in this study are listed in Table 1. *Acidovorax citrulli* strains were routinely grown at 28 °C in nutrient broth (NB; Difco Laboratories, Detroit, MI, USA), NA (NB containing 15 g/L agar) or Lysogeny Broth (LB; Difco Laboratories). XVM2 minimal medium, which resembles to some extent the plant apoplast environment [26], was used for growth curve experiments and for biofilm formation and lipolytic assays (see below). *Escherichia coli* strains were cultured in LB at 37 °C. Antibiotics were added at the following concentrations: ampicillin (Ap), 100 μg/mL; kanamycin (Km), 50 μg/mL; and gentamicin (Gm), 10 or 30 μg/mL (for *E. coli* and *A. citrulli*, respectively).

### 2.2. Molecular Biology Techniques

Genomic DNA was extracted with the GeneElute Bacterial Genomic DNA Kit (Sigma-Aldrich, St. Louis, MO, USA). Plasmids were extracted using the BioNeer AccuPrep Plasmid Mini Extraction kit (Daejeon, Republic of Korea). For preparation of cDNA, RNA extractions from NB-grown bacteria were carried out using TRI Reagent (Sigma-Aldrich). Then, genomic DNA was eliminated using DNA-free DNase (Ambion, Austin, TX, USA). cDNA was generated using random primers with the High Capacity cDNA Reverse Transcription kit (Applied Biosystems, Foster City, CA, USA). Each sample contained 1 µg RNA in 20 µL of a reaction mix. All enzymes and kits were used according to the manufacturer’s instructions. The primers used in this study were purchased from Sigma (Rehovot, Israel) and are listed in Table S1.

### 2.3. Isolation of the A. citrulli M6-lip1^-^ Mutant from a Transposon Mutant Library

The M6-*lip1*^-^ mutant was screened out of a random transposon library that contains about 10,000 mutants, and was generated in the background of *A. citrulli* M6 using the Ez-Tn5 kit (Epicentre, Madison, WI, USA), as described [11,16]. Mutants altered in twitching motility were directly screened on NA/Km selection plates by the naked eye, and verified by colony microscopy observations using an Axio Scope light microscope (Carl Zeiss, Jena, Germany) equipped with a DXM1200F digital camera (Nikon, Tokyo, Japan). The M6*-lip1^-^* mutant was tested by Southern blot hybridization to verify insertion of the EZ-Tn5 cassette, using the ECL Direct Nucleic Acid Labeling and Detection System (Amersham Biosciences, Buckinghamshire, UK) according to the manufacturers’ instructions, and using a region of the EZ-Tn5 cassette as probe [11]. The insertion site of the EZ-Tn5 cassette was identified following sequencing of the mutant genome by Illumina MiSeq at the Center for Genomic Technologies of the Hebrew University of Jerusalem. Quality trimming and genome assembly were conducted as described [14].

### 2.4. Sequence Analyses and Homology Modeling

BlastN and BlastP analyses were conducted using the National Center for Biotechnology Information (NCBI) Blast server (https://blast.ncbi.nlm.nih.gov/Blast.cgi, accessed on 10 December 2021). BlastP was also conducted against the UniProtKB/SwissProt database at the UniProt server (http://www.uniprot.org/blast/, accessed on 10 December 2021). Analysis of conserved domains was conducted using the Conserved Domains server (https://www.ncbi.nlm.nih.gov/Structure/cdd/wrpsb.cgi, accessed on 10 December 2021; [31]) with default parameters. A three dimensional (3D) homology modeling of Lip1 was generated using the I-TASSER server (https://zhanggroup.org/I-TASSER/, accessed on 15 April 2020) [32], using default parameters with no additional restraints. The model chosen for analysis was model1, which had a confidence score of −0.93. Visualization of protein structures and 3D figures were created using Discovery Studio 4.5 Visualizer (Dassault Systèmes Biovia, San Diego, CA, USA). Signal peptide prediction of Lip1 was done with Pred-Tat (http://www.compgen.org/tools/PRED-TAT/, accessed on 1 March 2022) [33] and Phobius (http://phobius.sbc.su.se/, accessed on 1 March 2022) [34].

### 2.5. Construction of Plasmids for Complementation and Assessment of Lip1 Secretion

**Figure 1:**
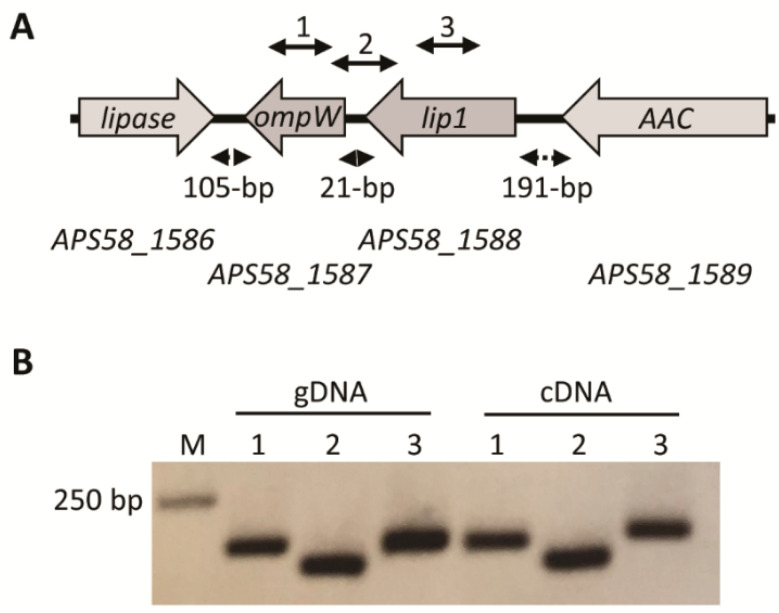
The *lip1-ompW* operon. (**A**) Schematic organization of the operon and neighboring genes according to the annotation of the *Acidovorax citrulli* M6 genome (GenBank accession CP029373.1). Grey arrows represent the open reading frames (ORFs) and transcription direction of the following genes (numbers between parentheses indicate the position of start and stop codons in the *A. citrulli* M6 chromosome): *APS58_1586*, hypothetical protein but having high similarity to GDSL lipases (1,779,911–1,780,861); *APS58_1587*, outer membrane protein OmpW (1,780,966–1,781,658, complement); *APS58_1588*, Lip1 (1,781,679–1,782,830; complement); and *APS58_1589*, D-alanyl-D-alanine carboxypeptidase (AAC; 1,783,021–1,784,559; complement). Dashed double arrows indicate the distance between the ORFs. Solid double arrows above the genes indicate the location of PCR targets for cDNA amplification (see Table S1). (**B**) Polymerase chain reaction (PCR) using *A. citrulli* M6 genomic DNA (gDNA), or cDNA synthesized from RNA extraction from an overnight NB-grown culture of *A. citrulli* M6. PCR reactions were conducted with primers described in Table S1 and the lane numbers correspond to the solid double arrows in panel A: (1) primers ompW_F and ompW_R for amplification of an internal fragment of *ompW*; (2) primers 21between_F and 21between_R for amplification of a region containing part of the *lip1* and *ompW* ORFs and their intergenic region; and (3) lip1_F and lip1_R for amplification of an internal fragment of *ompW*. Negative controls with no reverse transcriptase were used to verify that RNA samples do not contain genomic DNA contamination, and did not yield PCR products.

Several complementation strains were created on the background of the *A. citrulli* M6*-lip1*^-^ mutant. The first complementation strains were M6-comp-lip1 and M6-comp-ompW, carrying plasmid pBBR1-MCS-5 with the complete ORF of *lip1* or *ompW* (plasmids pBBRlip1 and pBBRompW, respectively; Table 1). In both cases, the constructs included a 250-bp region upstream of the *lip1* ORF, thus carrying the promoter region of the *lip1-ompW* operon (Figure 1A). These plasmids were generated using the restriction free (RF) cloning method (www.rf-cloning.org using primers that were designed based on the M6 genome annotation (GenBank accession CP029373.1) and are listed in Table S1. We first used the RF primers lipComp_F and lipComp_R to clone the promoter region and the *lip1* ORF in pBBR1-MCS-5 generating pBBRlip1. Further, the resulting plasmid was used to generate pBBRompW, by replacement of the *lip1* ORF by the *ompW* ORF using RF primers ompWcomp_F and ompWcomp_R. To assess whether Lip1 is secreted in a type II secretion-dependent manner, we generated plasmids pBBR1lip1-HA and pBBRlip1_35-383_-HA, carrying the complete ORF of *lip1* or the *lip1* ORF without the predicted, N-terminal signal peptide, respectively. In both cases, the ORFs were fused to the HA epitope at their C-terminus (Table 1 and Table S1). All PCR amplifications were conducted using High-Fidelity DNA polymerase Phusion (New England Biolabs, Beverly, MA, USA). The resulting plasmids were transformed into *E*. *coli* S17-1 λpir as described [11]. The plasmids were extracted and the cloned fragments were verified by sequencing at Hy Laboratories Ltd. (Rehovot, Israel). Conjugations with *A. citrulli* strains were performed by bi-parental mating as described [11]. Transconjugants were selected on the basis of Gm resistance and verified by PCR.

### 2.6. Transmission Electron Microscopy (TEM) Observations

TEM was used to visualize T4P and polar flagella in *A. citrulli* M6-*lip1*^-^ and wild-type M6. Bacterial samples were prepared from 48-h-old, NA-grown colonies. A 5-µL sterile water drop was placed on a selected colony for 2 min, then a 300 mesh carbon grid was placed on the top of each colony for 30 s and the grid was negatively stained in a 1% uranyl drop for 10 s. The grids were examined with a FEI Tecnai-12 electron microscope (FEI, Eindhoven, The Netherlands) equipped with a F224HD 2k × 2k CCD camera (TVIPS, Gauting, Germany).

### 2.7. Growth Curves and Biofilm Formation Assays

Growth of *A. citrulli* M6 and M6-*lip1*^-^ was assessed by incubation at 28 °C in rich (NB) and minimal (XVM2) media for 48 h. Bacteria were grown in 96-well polystyrene microplates (Nunc, Roskilde, Denmark; 100 µL of media in each well), in an Infinite F200 plate reader (Tecan, Männedorf, Switzerland), with linear shaking for 15 s every 30 min, and optical density was measured at OD_600_. Each experiment included 24 replicates per strain/medium. Biofilm assays were performed as described [35]. Briefly, overnight cultures of *A. citrulli* were diluted in a 1:100 ratio with fresh XVM2 media. Then, 200 μL bacterial suspensions were transferred into wells of 96-well polystyrene microplate (Nunc) and incubated at 28 °C for 48 h. The media were discarded and wells were washed twice with sterile distilled water (SDW). Two hundred microliters of 0.1% crystal violet were then added to each well and the plate was incubated at room temperature for 30 min. The crystal violet solution was discarded and the wells were washed gently with SDW (twice). Then 200 µL of 95% ethanol were added to the wells to solubilize the stained biofilms. After 2 h of incubation at room temperature the optical density of each well was measured at OD_595_ using the Infinite F200 plate reader.

### 2.8. Measurement of Twitching Haloes

*Acidovorax citrulli* strains were grown at 28 °C for 48 h on NA plates, and twitching haloes around the colonies were measured using EVOS microscope and software (Thermo Fisher Scientific, Waltham, MA, USA). In each experiment, twitching halo lengths of 30 colonies per strain were measured.

### 2.9. Virulence Assays

Seed transmission experiments were performed as described [11] with minor modifications. *Acidovorax citrulli* strains were grown on NA for 48 h, resuspended from plates with SDW, adjusted to an OD_600_ of 0.2 [about 10^8^ colony forming units (CFU)/mL] using a Helios Gamma spectrophotometer (Thermo Electron Corp., Rochester, NY, USA), and then diluted to 10^6^ CFU/mL. Inoculum concentrations were verified by dilution plating. Melon (*Cucumis melo*) cv. Ophir (Zeraim Gedera, Kibutz Revadim, Israel) seeds were placed into 50 mL-Falcon tubes containing 20 mL bacterial suspensions and incubated at room temperature for 2 h with gentle agitation. The bacterial suspensions were discarded, and the seeds were dried under a laminar hood and sown in 11-cm diameter poly pots (Tefen, Nahsholim, Israel) containing a peat-based commercial soil mixture (Shacham Givat Ada, Givat Ada, Israel). The pots were kept in a greenhouse at 26–28 °C. Disease severity was evaluated 10 days after sowing, using a 0–7 scale (0, healthy plants; 7, highest disease severity), which is based on the percentage of the foliage weight of infected plants relative to non-infected controls [36]. For infiltrations of *Nicotiana benthamiana* leaves, *A. citrulli* strains were grown on LB medium with the appropriate antibiotics at 28 °C for 24 h. Bacterial cells were collected by centrifugation (4500× *g*, 5 min), resuspended in a 10 mM MgCl_2_ solution to an OD_600_ of 0.6, and infiltrated with a needleless syringe into the abaxial part of the leaves of 4-week-old *N. benthamiana* plants. Symptoms were observed after 36 h.

### 2.10. Swimming Motility Assays and Measurement of Swimming Speed

Swimming motility assays were performed on soft NA plates, containing 0.3% agar as described [11]. Briefly, cells from single NA-grown colonies were transferred to the center of soft NA agar plates using a toothpick. The plates were incubated at 28 °C and swimming haloes were measured after 48 h. Swimming speed of *A. citrulli* cells was measured after overnight growth in NB at 28 °C, using an Eclipse T1-E inverted microscope equipped with a Digital Sight cooled monochrome CCD camera (Nikon), at a magnification of ×100 with BX51 phase-contrast. Time-lapse pictures were taken and swimming speed was calculated using FIJI, an open source platform for biological image analysis [37]. In each experiment swimming speed averages were calculated from 150 cells per strain.

### 2.11. Lipolytic Activity Assays

Assessment of lipolytic activity by *A. citrulli* cultures was carried out as described [23] with few modifications. Briefly, M6, M6-*lip1*^-^ and M6-comp-lip1 strains were grown for 48 h at 28 °C with shacking (200 rpm) in 5 mL XVM2 medium. Cells were pelleted by centrifugation (4500× *g*, 5 min, 4 °C; twice). The precipitated cells were resuspended in SDW, serially diluted and plated on NA plates for CFU counts. The supernatants were collected and assessed fluorometrically for quantitative lipolytic activity using the lipase substrate 4-methylumbelliferyl oleate (MUO; Sigma-Aldrich) [38]. One-milliliter supernatant samples were used with measurements being taken every 10 min, from time “zero” (just before addition of MUO) for 90 min. Standard curves were prepared using different concentrations of the fluorescent standard 4-methylumbelliferine (MU; Sigma-Aldrich).

### 2.12. Secretion Assays

Secretion assays were performed as described by Bernal et al. [39]. Briefly, bacteria were grown on LB with appropriate antibiotics at 28 °C for 24 h. Bacterial pellets were collected by centrifugation (10,000× *g*, 20 min; 3 times), normalized and added directly to 1× Laemmli buffer. The supernatants were collected and precipitated with trichloroacetic (TCA) acid overnight and then washed with acetone and resuspended in 1× Laemmli buffer. Proteins were separated by SDS–polyacrylamide gel electrophoresis containing 15% (*w*/*v*) acrylamide and electro-transferred to 0.45 µm-nitrocellulose membranes (Cytiva, Marlborough, MA, USA) using a Trans-Blot Turbo transfer system (Bio-Rad, Hercules, CA, USA). Immunodetection was performed using monoclonal antibodies directed against the HA epitope (Cell Signalling Technology, Danvers, MA, USA). The secondary antibody, anti-rabbit IgG HRP-linked (Cell Signalling Technology), was detected with the Immobilon Forte HRP substrate (Merck Millipore, Burlington, MA, USA) using an Odyssey XF Imaging System (Li-Cor, Lincoln, NE, SA, USA).

### 2.13. Statistical Analysis

Quantitative assays were analyzed by Tukey–Kramer test for mean comparison in conjunction with analysis of variance (ANOVA) using JMP software (SAS Institute Inc., Cary, NC, USA).

## 3. Results

### 3.1. Identification of an Acidovorax citrulli M6 Mutant with Increased Twitching Haloes

*Acidovorax citrulli* cells are motile on solid surfaces by means of T4P-mediated twitching motility. In this bacterium, twitching haloes around colonies growing on nutrient agar (NA) can be easily visualized by the naked eye after ~72 h of growth at 28 °C [11]. We exploited the simplicity of this phenotype to screen a library of about 10,000 EZ-Tn5 mutants in the background of the group I model strain, M6. As aforementioned, we identified fifty mutants impaired in twenty different genes that lacked twitching ability [16]. In these screens we also identified a single mutant, which was later named M6-*lip1^-^* (see below), that produced twitching haloes substantially larger than those generated by wild-type M6 colonies.

Southern blot analysis supported that the transposon was inserted only once in the M6-*lip1^-^* genome (Figure S1). To identify the site of transposon insertion, we tried a plasmid rescue approach [11,16] but in the case of this mutant we were not able to get any plasmid following digestion with several restriction enzymes. As an alternative approach, we sequenced the mutant genome by Illumina MiSeq. Following its assembly and comparison with the M6 genome (GenBank accession CP029373.1), we detected a single insertion of the EZ-Tn5 cassette, thus confirming Southern blot results. The transposon disrupted the open reading frame (ORF) of gene *APS58_1588* (Figure 1A).

The *APS58_1588* gene has an ORF of 1152 base pairs, encoding a 383 amino-acid protein with a calculated molecular weight of 38.8 KDa. Although this gene is annotated as encoding a hypothetical protein, BlastN against the NCBI nucleotide collection (nr/nt) matched with genes that were mostly annotated as phospholipases. The highest hits were genes from other *A. citrulli* strains (at 100% identity) and from strains of two other plant-pathogenic *Acidovorax* species, *A. avenae* and *A. cattleyae* (at 87 to 89% identity). Relatively high identity (75%) was also found for genes from two strains of *Melaminivora* sp., which like *A. citrulli*, belong to the *Comamonadaceae* family. Similarly, BlastP analysis of the *APS58_1588* product against the NCBI Protein Reference Sequences’ database matched with proteins from various plant-pathogenic *Acidovorax* species at full coverage. The highest identities were for proteins from *A. citrulli* (100%), *A. avenae* (89–90%), *A. cattleyae* (90%), *A. oryzae* (89%) and *A. konjaci* (82%). Relatively high levels of protein identity were found for other *Comamonadaceae* members including *Diaphrobacter polyhydroxybutyrativorans* (77% identity), *Melaminivora* sp. (67%), *Simplicispira psychrophila* (63%) and *Delftia acidovorans* (62%). Similar to the BlastN results, the BlastP hits were mostly annotated as phospholipases, although some were annotated as lipases or hypothetical proteins.

**Figure 2:**
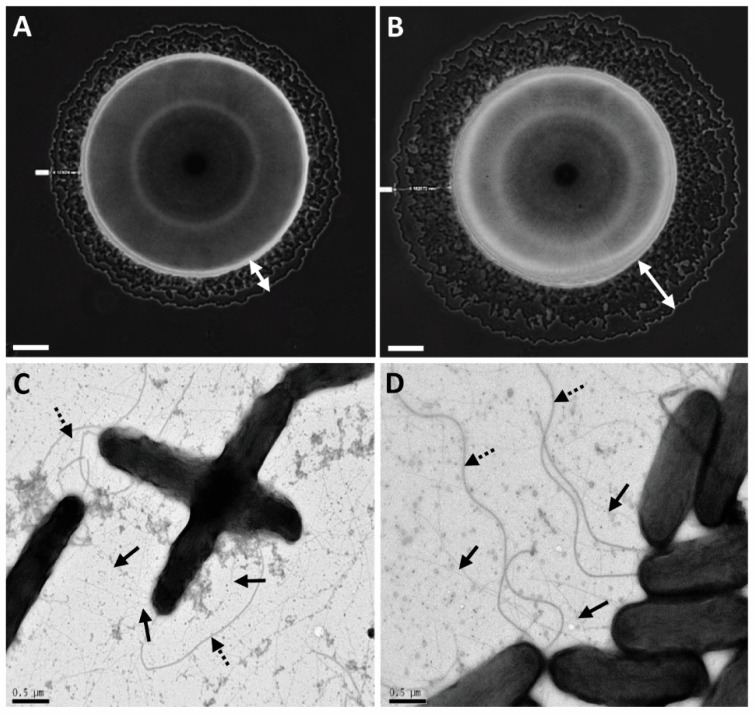
Twitching motility and type IV pilus (T4P) formation of *A. citrulli* M6 and an M6 mutant disrupted in gene *APS58_1588* (M6*-lip1*^-^). Images of bacterial colonies of M6 (**A**) and M6-*lip1*^-^ (**B**) seen by light microscopy after 48 h of growth on NA plates at 28 °C. Typical haloes surrounding the bulk colonies (indicated by the white double arrows) are formed by bacteria migrating via twitching motility. Pictures were taken using an Axio Scope light microscope equipped with a DXM1200F digital camera. Transmission electron microscopy observations of M6 (**C**) and M6-*lip1*^-^ (**D**). The strains were observed in a FEI Tecnai-12 electron microscope after growth on NA at 28 °C for 48 h. Solid and dashed arrows indicate polar flagellum and T4P, respectively. Bars at the bottom of each panel: 100 µm for (**A**,**B**); and 0.5 µm for (**C**,**D**).

Further observations under a light microscope revealed that differences between M6 and M6-*lip1^-^* haloes around the colonies were clearly visible after 48 h of growth (Figure 2A,B). Wild-type and mutant strains did not differ in the production of T4P and polar flagella, as observed by TEM (Figure 2C,D). Furthemore, no differences were observed between the strains in their growth ability in rich (NA) and minimal (XVM2) media, and in biofilm formation ability (Figures S2 and S3, respectively).

### 3.2. Sequence Analyses of APS58_1588 Support It Encodes a Lipolytic Enzyme from the GDSL Family

Although *APS58_1588* and most homologous genes are annotated as phospholipases, analysis of the *APS58_1588* product at the NCBI Conserved Domains (CD) server [31] suggested this gene encodes a lipase belonging to the triacylglycerol lipase-like subfamily (cd01847) of the SGNH hydrolase superfamily (cluster cl01053). One of the representatives of this subfamily is a protein annotated as lipase 1 (Lip1) from *Photorhabdus luminescens* (GenBank locus LIP1_PHOLU). Moreover, BlastP analysis of APS58_1588 against the UniProtKB/SwissProt database revealed *P. luminescens* Lip1 as its highest hit (though at only 23% identity for a query coverage of 89%). Based on this finding, on lipolytic assays of mutant and wild-type supernatants (see below), and on the fact that, to the best of our knowledge, this is the first lipase gene to be characterized in *A. citrulli*, we named the *APS58_1588* gene, *lip1*.

**Figure 3:**
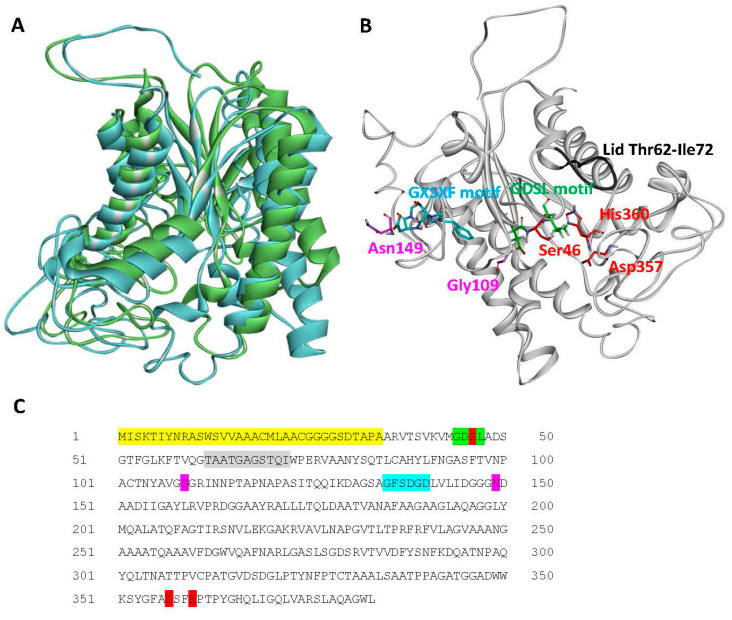
Structural analysis of *A*. *citrulli* M6 Lip1. (**A**) Structural alignment between Lip1 (blue ribbon), and the passenger domain of the autotransporter EstA from *Pseudomonas aeruginosa* (green ribbon; PDB code 3KVN). (**B**) Predicted structural model of Lip1 generated by I-TASSER, and (**C**) Lip1 sequence. Conserved residues and motifs are highlighted and shown in sticks as follows: red, catalytic triad; green (and red for Ser46), GDSL motif; blue, GXSXG motif; purple, oxyanion hole residues; and grey, putative lid loop (ribbon only). The secretion signal peptide as predicted by Pred-Tat and Phobius is highlighted yellow in the protein sequence.

We generated a 3D model of Lip1 using the I-TASSER server [32]. High similarity was found between Lip1 and the passenger domain of the autotransporter esterase EstA from *Pseudomonas aeruginosa* [40,41]. EstA is a member of the GDSL family of lipolytic enzymes. It also belongs to the autotransporter (type V secretion) protein family, being composed of two domains, the N-terminal passenger domain that also harbors the enzymatic activity, and the C-terminal domain that forms a β-barrel pore in the outer membrane, through which the passenger domain is secreted to the cell surface [40,42,43,44]. A structural alignment of Lip1 and the passenger domain of EstA are shown in Figure 3A. Sequence and structural analyses revealed that Lip1 contains the conserved catalytic triad residues, Ser46-Asp357-His360 (Figure 3B,C) in positions that are relatively similar to those found in *P. aeruginosa* EstA. In addition, typical conserved motifs in members of the GDSL family of lipolytic enzymes- GDSL (residues 44–47) and GXSXG (residues 135–139)- are found in Lip1 (Figure 3B,C). The GXSXG motif is referred to as the nucleophile elbow [45], which allows the activity of the catalytic triad. Based on structure similarity with the passenger domain of *P. aeruginosa* EstA, and on information available for the lid structure [46], we hypothesize that the lid region is a loop comprising residues Thr62-Ile72 (Figure 3B). Additional analysis using the CD server pointed at Gly109 and Asn149, which together with Ser46 of the catalytic triad and the aforementioned GDSL motif, could form the oxyanion hole of the active site (Figure 3B).

### 3.3. The lip1 Gene Is Part of an Operon with ompW

In *A. citrulli* M6, the *lip1* gene appears to be part of an operon with *APS58_1587* (Figure 1A). This gene encodes an outer membrane protein, OmpW, which in other bacteria was suggested to be involved in the transport of small hydrophobic molecules [47,48]. To assess whether *lip1* and *ompW* indeed comprise an operon, primers were designed to amplify internal regions of the two ORFs as well as a region spanning the two genes and their intergenic region (Figure 1A; Table S1). *Acidovorax citrulli* M6 was grown overnight in NB medium, after which RNA was extracted. cDNA synthesized from the RNA extract was used as a template for PCR reactions. The results confirmed that both genes are expressed under tested conditions, and that *lip1* and *ompW* are indeed part of an operon (Figure 1B). Remarkably, an RNA-Seq approach that measured expression of M6 genes in the apoplast-mimicking medium XVM2, showed that both genes are expressed in this medium, and confirmed their operon organization as cDNA reads were detected that spanned the *lip1* and *ompW* ORFs [10; RNA-Seq data available under NCBI BioProject PRJNA565338].

### 3.4. Increased Twitching Haloes in the lip1^-^ Mutant Is due to lip1 Disruption

**Figure 4:**
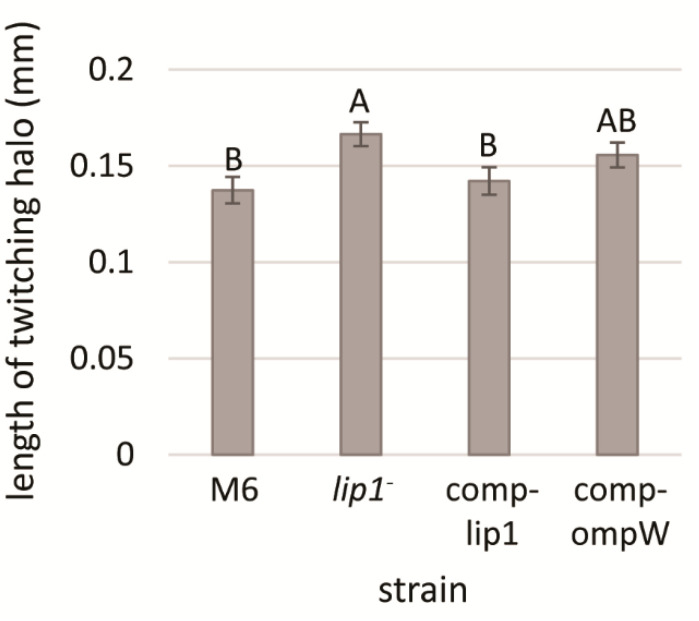
Length of twitching haloes of *A. citrulli* M6, the M6-*lip1*^-^ mutant, and M6-*lip1*^-^ carrying plasmid pBBR-MCS-5 expressing *lip1* or *ompW* (M6-comp-lip1 and M6-comp-ompW, respectively). Bacteria were grown for 48 h on NA plates at 28 °C, and twitching haloes around the colonies were measured from 30 colonies per strain. Data represent averages and standard errors (SE) from one experiment, out of three with similar results. Different letters indicate significant differences (*p* < 0.05) among treatments by Tukey–Kramer and ANOVA.

To assess whether the increased twitching haloes in the M6-*lip1*^-^ mutant is a direct result of disruption of the *lip1* gene or a polar effect of the mutation on *ompW*, we generated two complementation strains in the background of the mutant: M6-comp-lip1 and M6-comp-ompW. These strains were generated by transforming the M6-*lip1*^-^ mutant with plasmids pBBRlip1 and pBBRompW carrying *lip1* and *ompW*, respectively, under the control of the *lip1-ompW* operon promoter (Table 1). Complementation of the mutant with pBBRlip1 restored the wild-type phenotype in terms of twitching halo size (Figure 4). The M6*-lip1*^-^ mutant carrying pBBRompW yielded intermediate values of twitching haloes between the wild-type and the mutant strain; however, they did not significantly differ from those recorded for the M6-*lip1*^-^ mutant (Figure 4). Overall, these results support that the increased twitching haloes observed around M6-*lip1*^-^ colonies are due to disruption of the *lip1* gene.

### 3.5. The M6-lip1^-^ Mutant Has Reduced Virulence Relative to Wild-Type M6

**Figure 5:**
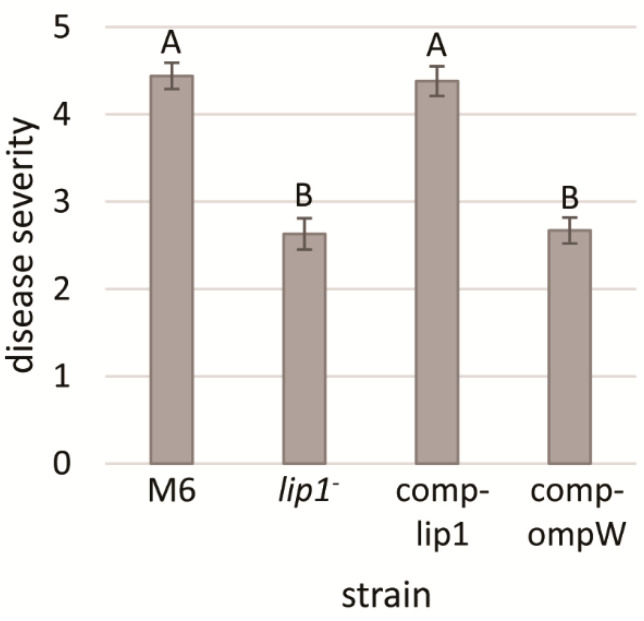
Seed transmission assays of melon inoculated with *A. citrulli* M6, the M6-*lip1*^-^ mutant, and M6-*lip1*^-^ carrying plasmid pBBR-MCS-5 expressing *lip1* or *ompW* (M6-comp-lip1 and M6-comp-ompW, respectively). Melon cv. Ophir seeds were inoculated with bacteria at 10^6^ CFU/mL, sown in a peat-based commercial soil mixture and maintained in the greenhouse at 26–28 °C for 10 days. Disease severity was determined using a 0–7 scale (zero, healthy; seven, highest severity) based on the percentage of the foliage weight of infected plants relative to non-infected controls. Data represent averages and SE from one experiment (15 plants per treatment), out of three with similar results. Different letters indicate significant differences (*p* < 0.05) among treatments by Tukey–Kramer and ANOVA.

*Acidovorax citrulli* is a seed-borne pathogen and melon seedlings are highly susceptible to the pathogen [2]. Therefore, we carried out seed transmission assays to assess whether *lip1* contributes to *A. citrulli* virulence. Disease severity was evaluated 10 days after inoculation (d.a.i), using a scale based on seedling weight [36]. Seedlings emerging from seeds that were inoculated with the M6-*lip1*^-^ mutant exhibited significantly (*p* < 0.05) lower disease severity as compared with seedlings emerging from M6-inoculated seeds (Figure 5). Complementation of the mutant by expression of intact *lip1* gene in plasmid pBBRlip1 (M6-comp-lip1 strain) restored wild-type levels of virulence. In contrast, wild-type levels of virulence could not be restored when the mutant carried pBBRompW, expressing the *ompW* gene (Figure 5). These results demonstrate that *lip1* significantly contributes to *A. citrulli* M6 virulence.

### 3.6. The M6-lip1^-^ Mutant Swims Slower than the Wild-Type Strain

**Figure 6:**
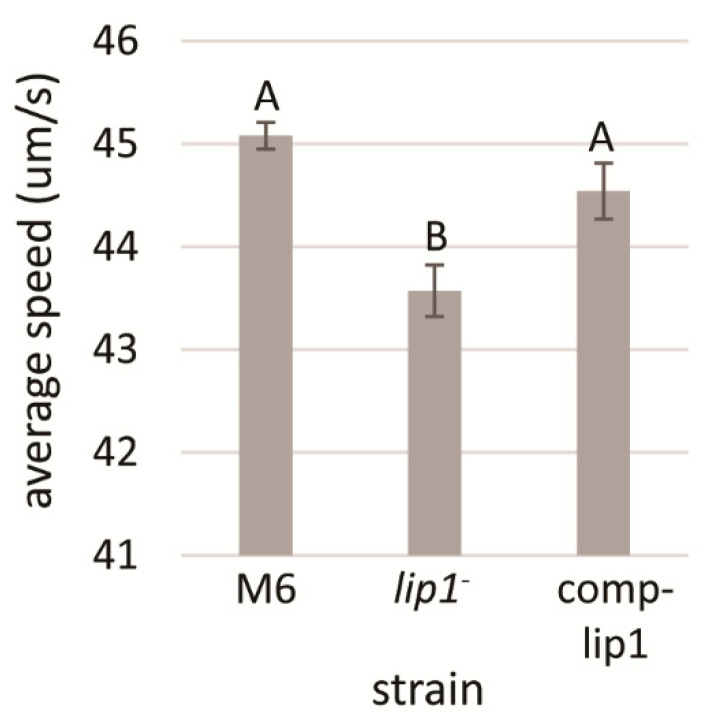
Analysis of swimming speed of *A. citrulli* M6, the M6-*lip1*^-^ mutant and the *lip1* complemented mutant (M6-comp-lip1). Swimming speed measurements were calculated from NB-overnight cultures, as average of 150 bacterial cells for each strain, using a Nikon Eclipse T1-E inverted microscope equipped with a Digital Sight cooled monochrome CCD camera. Time-lapse pictures were taken and swimming speed was calculated using FIJI. Data represent averages and SE from one experiment out of three with similar results. Different letters indicate significant differences (*p* < 0.05) among treatments by Tukey–Kramer and ANOVA.

Polar flagellum is an important virulence factor of *A. citrulli* [49]. Therefore, we assessed whether the *lip1*^-^ mutant has altered flagellum-mediated swimming motility relative to wild-type M6. No differences in this trait could be observed between the strains in swimming motility assays performed on soft agar plates (NA containing 0.3% agar) after 48 h of incubation at 28 °C. Since this assay may not be sufficiently sensitive, we carried out microscope monitoring of swimming speed of both strains after overnight growth of bacteria in NB. Time laps microscopic tracking and calculations of swimming speed revealed that, under tested conditions, *M6-lip1*^-^ cells had significantly (*p* < 0.05) lower swimming speed than wild-type cells. In contrast, the *lip1* complemented strain (M6-comp-lip1) did not significantly differ from the wild-type strain in this trait (Figure 6).

### 3.7. Lip1 Possesses Lipolytic Activity

**Figure 7:**
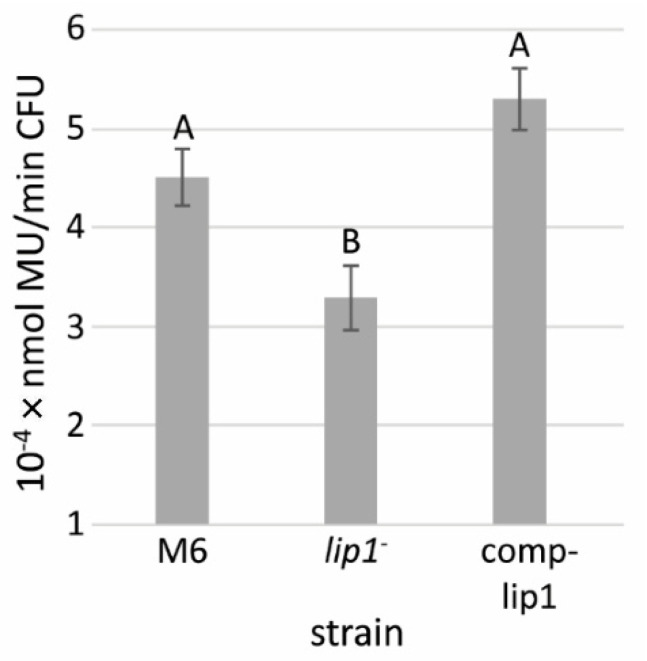
Lipolytic activity of supernatants from *A. citrulli* M6, the M6-*lip1*^-^ mutant, and M6-*lip1*^-^ carrying plasmid pBBR-MCS-5 expressing *lip1* (M6-comp-lip1). The strains were grown in XVM2 medium for 48 h at 28 °C with shacking (200 rpm). The supernatants were collected and used for measurement of lipolytic activity using 4-methylumbelliferyl oleate (MUO) as a substrate. Data represent averages and SE from three experiments with similar results (five replicates per strain in each experiment). Different letters indicate significant differences (*p* < 0.05) among treatments by Tukey–Kramer and ANOVA. MU, 4-methylumbelliferine; CFU, colony forming units.

Sequence analyses strongly indicated that Lip1 belongs to the GDSL family of lipases/esterases. We compared the lipolytic activities of the supernatants of XVM2-grown M6-*lip1*^-^, wild-type and *lip1* complemented strains, using the lipase substrate, 4-methylumbelliferyl oleate (MUO). These experiments showed a significantly (*p* < 0.05) lower lipolytic activity in the supernatants of M6-*lip1*^-^ cultures relative to those of wild-type and complemented strains (Figure 7). Lipolytic activity was still detected in the mutant supernatant, probably due to the activity of other lipolytic enzymes.

### 3.8. Lip1 Is Secreted in a Type II Secretion-Dependent Manner

In *Proteobacteria*, lipolytic enzymes are commonly secreted by type II secretion (T2S) systems, which are generally referred to as the main branch of the general secretory pathway (Gsp; Sec-pathway) [50,51]. We analyzed the Lip1 sequence using the Pred-Tat server, which predicts N-terminal Sec-pathway or twin-arginine translocation (Tat) signal peptides with Hidden Markov models [33]. This analysis revealed the presence of a Sec-pathway signal peptide composed by the first 34 amino acids in the Lip1 N-terminus, with a reliability score of 0.997. According to this prediction, the cleavage site is between two alanine residues, Ala34 and Ala35 (Figure 3C). An identical prediction supporting the 34-amino acid-signal peptide in the N-terminus of Lip1 was obtained using the Phobius prediction tool [34].

**Figure 8:**
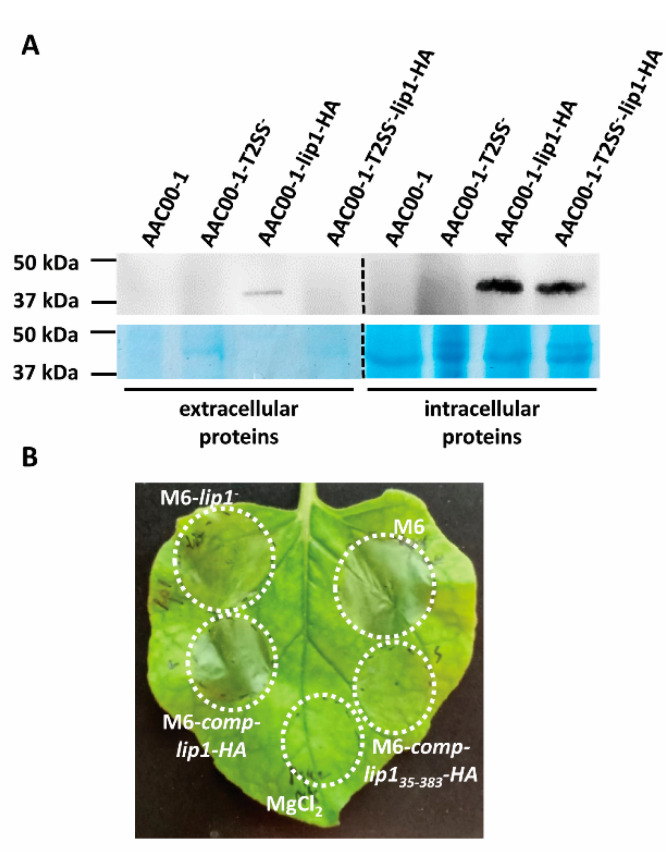
Type II secretion (T2S) of Lip1. (**A**) Western blot analysis of extracellular and intracellular fractions of *A. citrulli* strains (AAC00-1, wild-type; AAC00-1-T2SS^-^, T2S mutant) expressing or not recombinant Lip1-HA (see details in Materials and Methods). Representative images of one out of two experiments with similar results are shown. Dashed lines between extracellular and intracellular proteins indicate that the images were composed for comprehensive visualization of treatments. The original images are shown in Figure S4. (**B**) Infiltration of four-week-old *N. benthamiana* leaves with strains M6, M6-*lip1^-^* and M6-*lip1^-^* expressing full length Lip1-HA or the Lip1 ORF lacking the N-terminal secretion signal (strains M6-comp-lip1-HA and M6-comp-lip1_35-383_-HA, respectively). The picture was taken 36 h after infiltration and is representative of three independent experiments with similar results (with each experiment involving at least five leaves).

We introduced plasmid pBBRlip1-HA, carrying the *lipA* ORF fused to an HA tag, to *A. citrulli* AAC00-1, and to a mutant of this strain impaired in T2S [2,27]. Western blot analysis showed that while the LipA-HA recombinant protein was produced and detected in the intracellular fraction of both strains, the protein could be detected in supernatants of wild-type AAC00-1 cultures but not in those of the T2S mutant (Figure 8A and Figure S4). The LipA-HA signal was weaker in supernatants than in the intracellular fractions due to the lower concentration of proteins in the former.

*Nicotiana benthamiana* was recently demonstrated as a suitable surrogate host for investigation of *A. citrulli* pathogenicity [52]. We introduced pBBRlip1-HA in the M6-*lip1*^-^ mutant and used the resulting strain, M6-comp-lip1-HA, for leaf infiltration of *N. benthamiana* leaves, in comparison with wild-type M6 and M6-*lip1^-^* strains. While the mutant strain was severely compromised in its ability to induce symptoms in *N. benthamiana* leaves, strain M6-comp-lip1-HA performed as similar as the wild-type strain (Figure 8B). Remarkably, plasmid pBBRlip1_35-383_-HA carrying the lip1 ORF without the predicted signal peptide (Table 1), was not able to complement the M6-*lip1^-^* mutant for virulence in these assays (Figure 8B). Overall, our results confirm that Lip1 is secreted in a T2S-dependent manner, and this is required for wild-type levels of virulence of *A. citrulli*.

## 4. Discussion

Twitching motility is a flagellum-independent translocation mechanism that promotes bacterial movement on solid surfaces. This type of motility is mediated by type IV pili (T4P), hair-like polar appendages that are found in a wide range of bacteria belonging to the Proteobacteria, Cyanobacteria and Firmicutes [53,54]. Besides mediating twitching motility, T4P are involved in surface adhesion, colonization, biofilm formation, genetic material uptake and virulence [13,54,55]. Among plant-pathogenic bacteria, a significant contribution of T4P to virulence has been demonstrated mainly for vascular, xylem-colonizing bacteria [54]. In these pathogens, T4P may contribute to colonization and spread in the xylem vessels by promoting biofilm formation on the vessel surfaces as well as twitching motility in this niche [54].

Due to the importance of T4P and twitching motility for virulence of *A. citrulli* [11], we screened a mutant library of the model *A. citrulli* strain M6 for altered twitching phenotypes. Fifty mutants impaired in twenty different genes were detected in this screen that showed no twitching ability and compromised virulence [16]. Interestingly, one single mutant was found to display larger twitching motility haloes in comparison with the wild-type strain (Figure 2 and Figure 4). Sequence analyses of the disrupted gene in this mutant revealed it encodes a protein belonging to the GDSL family of lipases/esterases. Lipolytic assays of bacterial supernatants with the lipase substrate MUO confirmed that the mutated gene encodes a lipolytic enzyme (Figure 7), which we named Lip1. Remarkably, the *A. citrulli* M6-*lip1^-^* mutant was found to be significantly compromised in virulence relative to wild-type M6 (Figure 5 and Figure 8B). In *A. citrulli*, mutants damaged in twitching ability and/or T4P synthesis are also compromised in biofilm formation ability [11,12,16]. Here we show that under tested conditions, the M6*-lip1*^-^ mutant does not significantly differ from wild-type M6 in this trait (Figure S2).

The relationship between lipolytic enzymes and motility has been well investigated in the opportunistic pathogen *Pseudomonas aeruginosa*. Barker et al. [56] showed that a phospholipase, PlcB, is required for directed twitching motility in response to gradients of certain phospholipids. Wilhelm et al. [41] showed that a *P. aeruginosa* mutant defective in the esterase gene *estA*, was affected in twitching and swimming motilities as well as in biofilm formation ability. The involvement of EstA and the lipases LipA and LipC in twitching of *P. aeuriginosa* in response to the phospholipid phosphatidylethanolamine (PE) was further demonstrated by Miller et al. [57]. A *lipC* mutant of this bacterium was also affected in swimming motility [58]. Here we show that the *A. citrulli* M6-*lip1^-^* mutant has lower swimming speed than the wild-type strain (Figure 6).

Lipases are extracellular enzymes and must therefore be translocated through the bacterial membrane to find their way out of the cell [17]. In Gram-negative bacteria, most lipases/esterases are secreted via type I or type II secretion (T1S and T2S, respectively) systems, but an additional secretory pathway is the type V/autotransporter system [59]. The latter is the case of the aforementioned EstA esterase of *P. aeruginosa*, which is required for rhamnolipid production [41,60]. The crystal structure of EstA was solved by van den Berg [40]. Interestingly, structure prediction of *A. citrulli* Lip1 revealed that, among proteins with solved structures, the N-terminal passenger domain of EstA has the highest structural similarity to Lip1. Sequence analyses also revealed that Lip1 carries typical motifs of members of the GDSL family of lipases/esterases.

Two different prediction tools revealed that Lip1 has a predicted 34-amino acid signal peptide in its N-terminus (cleavage site between Ala34 and Ala35), which probably mediates its translocation through the cytoplasmic membrane via the general secretory pathway (Gsp; Sec-pathway). Since Lip1 does not possess an autotransporter β-barrel domain like EstA, we hypothesized that Lip1 is transferred to the periplasm via the Sec-pathway, and from the periplasm out of the cell, via T2S. This hypothesis was further validated using an *A. citrulli* mutant defective in T2S (Figure 8A). Remarkably, a neighbor gene of *lip1*, *APS58_1589* (Figure 1A), encodes D-alanyl-D-alanine carboxypeptidase (DD-CPase). DD-CPases are enzymes that belong to the penicillin-binding protein (PBP) family, some of which possess Ala-Ala endopeptidase activity [61]. Further investigation is needed to assess whether the *APS_1589* gene encodes a *bona fide* DD-CPase and if its product mediates cleavage of the Lip1 signal peptide.

Although Lip1 significantly contributes to the lipolytic activity of *A. citrulli*, the *M6-lip1^-^* mutant still retained this activity (Figure 7). This is probably due to the presence of additional lipolytic enzymes. Indeed, the annotated genome of *A. citrulli* M6 contains additional genes annotated as lipases, phospholipases and esterases. We cannot discard the possibility that some of these genes are misannotated, and that few other genes annotated as hypothetical proteins could in fact be encoding enzymes with lipolytic activity. For instance, similar to *lip1*, the closely located gene *APS58_1586* (Figure 1A) was also automatically annotated as encoding a hypothetical protein, however Blast analysis revealed it encodes a protein with high similarity to GDSL lipases.

We showed that in *A. citrulli* M6, *lip1* is located in an operon together with the *ompW* gene. This seems also to be the case for the sequenced group II strain of *A. citrulli*, AAC00-1 (GenBank accession NC_008752.1), where *lip1* and *ompW* correspond to genes *Aave_*4567 and *Aave_*4566, respectively. OmpW is a member of a major family of outer membrane proteins that are widespread in Gram-negative bacteria. These proteins have been proposed to form channels for uptake of small hydrophobic molecules [47,48]. Evidence from studies with several bacterial species support that OmpW proteins are involved in adaptive responses to a variety of stresses, including salinity, temperature and exposure to antibiotics [62,63,64]. In *Escherichia coli*, OmpW forms part of the colicin S4 receptor [65]. Here we showed that the twitching and virulence phenotypes that were altered in the *lip1*^-^ mutant could be restored to wild-type levels by *in trans* expression of *lip1*, but not of *ompW*. These results demonstrate that the observed phenotypes were a direct consequence of *lip1* disruption and not due to a polar effect on *ompW* expression, as sometimes observed in transposon-mediated mutagenesis, including with EZ-Tn5 [66]. With that said, it is yet to be elucidated whether there is a functional relationship between Lip1 and OmpW.

Relatively few studies have studied the contribution of lipolytic enzymes to virulence of plant-pathogenic bacteria. In this regard, some lipases/esterases were shown to be important virulence factors in *Burkholderia glumae* [22], *Xanthomonas oryzae* pv. *oryzae* [20,21], *Xanthomonas perforans* [23], *Xylella fastidiosa* [25] and *Pseudomonas syringae* pv。*actinidiae* [24]. Lipolytic enzymes also play a role in virulence of several fungal plant pathogens such as *Blumeria graminis* [67] and *Fusarium* spp. [68]. Overall, there is little understanding about mechanistic aspects associated with the contribution of lipolytic enzymes to pathogens’ virulence and fitness. Several mechanisms have been proposed, including the degradation of host cell membranes, nutrient acquisition by digestion of lipids, adhesion through release of free fatty acids, interference with the host immune system, inactivation of bactericidal lipids and increased competition with other microorganisms [23,67,68,69,70,71]. Here, we showed that the *A. citrulli M6-lip1*^-^ mutant has lower swimming motility speed than the wild-type strain. Since polar flagellum mutants of *A. citrulli* have been shown to be compromised in virulence [49], it could be that the alteration in swimming ability by the disruption of *lip1* could also be associated, at least partially, with the reduced virulence ability of this mutant.

## 5. Conclusions

In this study we reported the characterization of an *A. citrulli* mutant defective in *lip1*, a gene encoding a GDSL-lipolytic enzyme that is expressed in both rich and minimal media. To the best of our knowledge, this is the first report on the contribution of a lipolytic enzyme to virulence of a plant-pathogenic *Acidovorax* species. Further investigation is needed to elucidate the mechanisms by which Lip1 contributes to *A. citrulli* virulence, and to assess the importance of other lipolytic enzymes in the pathogenicity of this bacterium.

## Figures and Tables

**Table 1:** Strains and plasmids used in this study.

Strains/Plasmids	Characteristics *	Reference or Source
*Acidovorax citrulli*		
M6	Wild-type strain; Ap^R^	[6]
M6-*lip1^-^*	M6 Tn5 mutant disrupted in gene *APS58_1588* (*lip1*) by the insertion of EZ-Tn5 transposon; Ap^R^, Km^R^	This study
M6-comp-lip1	M6 *lip1*^-^ mutant carrying pBBRlip1 (*lip1* complemented strain); Ap^R^, Km^R^, Gm^R^	This study
M6-comp-ompW	M6 *lip1*^-^ mutant carrying pBBRompW; Ap^R^, Km^R^, Gm^R^	This study
M6-comp-lip1-HA	M6 *lip1*^-^ mutant carrying pBBRlip1-HA; Ap^R^, Km^R^, Gm^R^	This study
M6-comp-lip1_35-383_-HA	M6 *lip1*^-^ mutant carrying pBBRlip1_35-383_-HA; Ap^R^, Km^R^, Gm^R^	This study
AAC00-1	Wild-type strain; Ap^R^	[4]
AAC00-1-lip1-HA	AAC00-1 carrying pBBRlip1-HA; Ap^R^, Gm^R^	This study
AAC00-1-T2SS^-^	AAC00-1 mutant impaired in type II secretion (double Δ*gsp*G1/Δ*gsp*G2 mutant); Ap^R^	[27]
AAC00-1-T2SS^-^-lip1-HA	AAC00-1-T2SS^-^ carrying pBBRlip1-HA; Ap^R^, Gm^R^	This study
*Escherichia coli*		
S17-1 λ pir	λ lysogenic S17-1 derivative producing π protein for replication of plasmids carrying *oriR6K*; *recA pro hsdR* RP4-2-Tc::Mu-Km::Tn7 λ– pir	[28]
Plasmids		
pUC-4K	pUC4 derivative (pMB1 ori), containing the Km^R^ gene from Tn903 that was used to generate the EZ-Tn5 transposon for mutagenesis; Ap^R^, Km^R^	[29]
pMOD-3<R6Kγ*ori*/MCS>	transposon construction vector; Ap^R^	Epicentre, Madison, WI, USA
pBBR1-MCS-5	broad host range vector; Gm^R^	[30]
pBBRlip1	pBBR1-MCS-5 containing the promotor region (250-bp) of the *lip1-ompW* operon followed by the *lip1* open reading frame (ORF) from M6; Gm^R^	This study
pBBRompW	pBBR1-MCS-5 containing the promotor region (250-bp) of the *lip1-ompW* operon followed by the *ompW* ORF from M6; Gm^R^	This study
pBBRlip1-HA	pBBR1-MCS-5 containing the *lip1* ORF fused to the HA epitope at its C-terminus, under the control of the *lac* constitutive promoter; Gm^R^	This study
pBBRlip1_35-383_-HA	pBBR1-MCS-5 containing the *lip1* ORF without the N-terminal signal peptide (first 102 bp of the ORF) fused to the HA epitope at its C-terminus, under the control of the lac constitutive promoter; Gm^R^	This study

* Km^R^, Ap^R^ and Gm^R^ indicate kanamycin, ampicillin and gentamicin resistance, respectively.

## Data Availability

Not applicable.

## Supplementary Materials

The following supporting information can be downloaded at: https://www.mdpi.com/article/10.3390/microorganisms10051016/s1, Figure S1: Southern blot analysis for verification of the insertion of the EZ-Tn5 cassette in the chromosome of *Acidovorax citrulli* M6-*lip1^-^*; Figure S2: Growth curves of *A. citrulli* M6 and *lip1*^-^ mutant in rich and in minimal media; Figure S3: Quantification of biofilm formation of *A. citrulli* strains M6 and *lip1*^-^ mutant; Figure S4: Type II secretion of Lip1; Table S1: Sets of PCR primers used in this study.
