# Supplementary material for: The GDSL-Lipolytic Enzyme Lip1 Is Required for Full Virulence of the Cucurbit Pathogenic Bacterium Acidovorax citrulli
Source: Microorganisms. 2022 May 12;10(5):1016. doi: 10.3390/microorganisms10051016 (PMC9147443; doi:10.3390/microorganisms10051016)
Supplement: Supplementary file 1 [file microorganisms-10-01016-s001.zip › microorganisms-1711204-supplementary.pdf]

Supplementary information

The GDSL-lipolytic enzyme Lip1 is required for full virulence of the cucurbit pathogenic bacterium *Acidovorax citrulli*

Tally Rosenberg, Irene Jiménez-Guerrero, Dafna Tamir-Ariel, Tali Yarnitzky and Saul Burdman

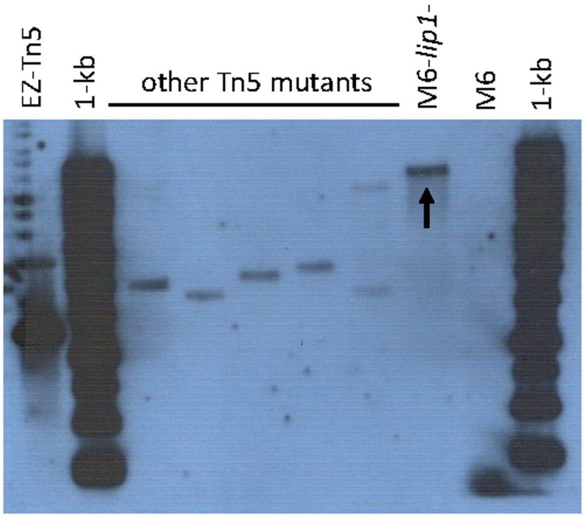

Figure S1. Southern blot analysis for verification of the insertion of the EZ-Tn5 cassette in the chromosome of *Acidovorax citrulli* M6-*lip1*$^{-}$. Hybridization was done with the ECL Direct Nucleotide Detection System (Amersham Biosciences) according to manufacturers' instructions, and using a labeled probe corresponding to the EZ-Tn5 cassette, as described [1]. Lanes from left to right: EZ-Tn5 DNA (positive control), 1-kb ladder, five (different) Tn5 mutants, M6-*lip1*$^{-}$, wild-type M6 (negative control), 1-kb ladder. The black arrow indicates a single detection of the EZ-Tn5 cassette in the M6-*lip1*$^{-}$ mutant (which was further confirmed by whole genome sequencing).

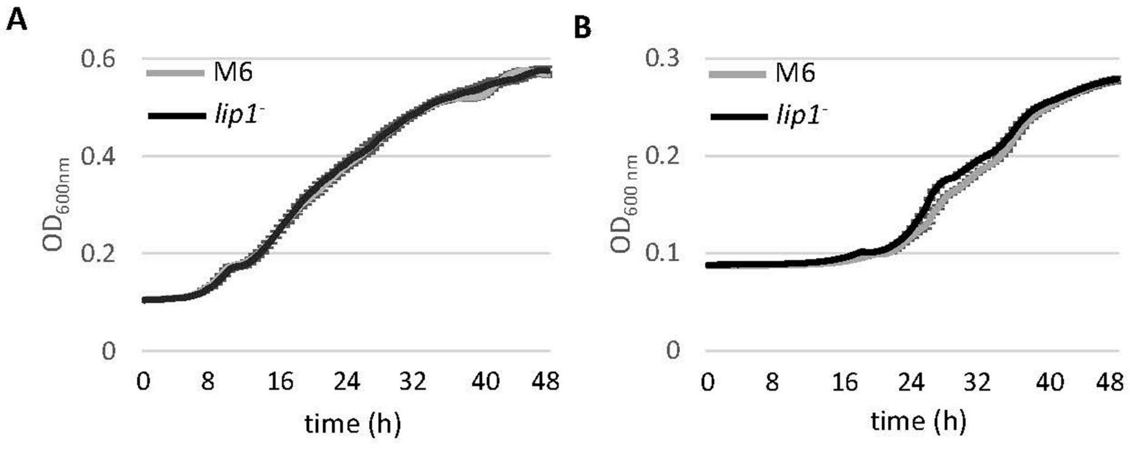

Figure S2. Growth curves of *A. citrulli* M6 and *lip1*$^{-}$ mutant in rich and in minimal media. The strains were grown for 48 h in rich [nutrient broth, NB; **(A)**] or minimal [XVM2 **(B)**] at 28 °C in an Infinite F200 plate reader (Tecan), with linear shaking for 15 s every 30 min. Results are from one representative experiment (24 replicates per strain), out of three with similar results.

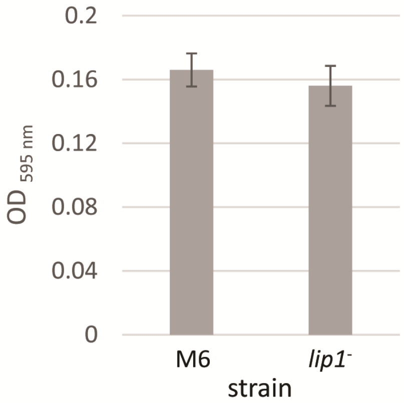

Figure S3. Quantification of biofilm formation of *A. citrulli* strains M6 and *lip1*- mutant. Cultures were grown in XVM2 medium for 48 h in 96-well polystyrene plates at 28 °C. Biofilm formed at the medium-air interface were stained using 0.1% crystal violet. Stained biofilms were resuspended in ethanol and quantitatively estimated by optical density measurements at $OD_{595\ nm}$. Data represent averages and standard errors of one experiment (10 replicates per treatment) out of three with similar results. No significant differences were found between the two strains by Tukey-Kramer test.

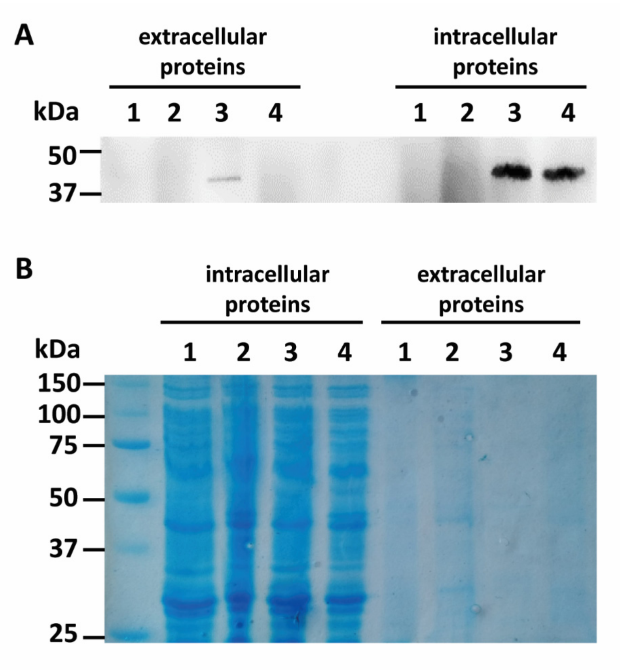

Figure S4. Type II secretion of Lip1. Original images of Western blot **(A)** and SDS-PAGE Coomassie staining **(B)** of extracellular and intracellular fractions of *A. citrulli* strains used for generation of Figure 8A in the manuscript. Experimental details are described in Materials and Methods. Strains: 1, wild type AAC00-1; 2; AAC00-1-T2SS- (T2S mutant); 3, AAC00-1-lip1-HA (AAC00-1 expressing HA-tagged Lip1); and 4, AAC00-1-T2SS--lip1-HA (AAC00-1-T2SS- expressing HA-tagged Lip1).

Table S1. Sets of PCR primers used in this study.

**Primers** *	**Primer sequence (5'-3')**	**Purpose and size of the amplified product**
lipComp_F	CGTAATACGACTCACTATAGGGCG AATTGTAGTTGATGCCGCCCGTGA ATGCG	Amplification of the *lip1* open reading frame (ORF) and its 250-bp upstream region carrying the *lip1-ompW* promoter (size: 1402 bp); used for construction of plasmid pBBRlip1 (see Table 1 and Materials and Methods).
lipComp_R	CCCTCACTAAAGGGAACAAAAGCT GGATGCTTGCGGCCTGCGGCGGT	
ompWComp_F	ATGGCGAACACTCCTATCCGCATC AGAACCGGTAGCCGAT	Amplification of the *ompW* ORF (size: 693 bp) for replacement of the *lip1* ORF in pBBRlip1, resulting in plasmid pBBRompW (see Table 1 and Materials and Methods).
ompWComp_R	CCCTCACTAAAGGGAACAAAAGCT GAATGGCTCGATTCCTCTCTTTC	
lip1_F	ATCCTTGATCTGCTGGGTGA	Amplification of an internal part of *lip1* (size: 153 bp); used for amplification of cDNA (target 3 in Fig. 3A).
lip1_R	AATTACAGCCAGACGCTGTG	
21between_F	CCCAGTTGTCGGTCACCAT	Amplification of a region spanning the *lip1* and *ompW* ORFs and their intergenic region (size: 109 bp); used for amplification of cDNA (target 2 in Fig. 3A).
21between_R	GTCAAGAGCGGCAACCTGT	
ompW_F	TCGTTGATGTTCCAGACGAA	Amplification of an internal part of *ompW* (size: 147 bp); used for amplification of cDNA (target 1 in Fig. 3A).
ompW_R	CGTGACCTACGCCAAGTTCT	
lip1HA_F	ATAGAATTCATGATCTCTAAAACC ATCTA	Amplification of the *lip1* ORF fused to the HA epitope sequence (size: 1179 bp); used for the generation of plasmid pBBRlip1-HA (see Table 1 and Materials and Methods).
lip1HA_R	ATAGGATCCTCAAGCGTAATCTGG AACATCGTATGGGTAGAGCCAGCC CGCCTGGGC	
lip1HAwoNt_F	ATAGAATTCATGGCGCGGGTGACT TCCGTG	Amplification of the *lip1* ORF without the signal peptide sequence fused to the HA epitope sequence (size: 1080 bp); used for the generation of plasmid pBBRlip1$_{35\text{-}383}$-HA (see Table 1 and Materials and Methods).
lip1HA_R	ATAGGATCCTCAAGCGTAATCTGG AACATCGTATGGGTAGAGCCAGCC CGCCTGGGC	

* F, forward; R, reverse.

References

1. Bahar, O.; Goffer, T.; Burdman, S. Type IV pili are required for virulence, twitching motility, and biofilm formation of *Acidovorax avenae* subsp. *citrulli*. *Mol. Plant-Microbe Interact.* **2009**, *22*, 909-920, doi:10.1094/mpmi-22-8-0909.
